# Aerobic exercise in obese diabetic patients with chronic kidney disease: a randomized and controlled pilot study

**DOI:** 10.1186/1475-2840-8-62

**Published:** 2009-12-09

**Authors:** David J Leehey, Irfan Moinuddin, Joseph P Bast, Shahzad Qureshi, Christine S Jelinek, Cheryl Cooper, Lonnie C Edwards, Bridget M Smith, Eileen G Collins

**Affiliations:** 1 Medicine Service, Veterans Affairs Hospital, Hines, IL, USA; 2 Research Service, Veterans Affairs Hospital, Hines, IL, USA; 3 Nutrition and Food Service, Veterans Affairs Hospital, Hines, IL, USA; 4 Spinal Cord Injury (SCI) QUERI and Center for Management of Complex Chronic Care, Veterans Affairs Hospital, Hines, IL, USA; 5 Department of Medicine, Loyola University Medical Center, Maywood, IL, USA; 6 Department of Medical-Surgical Nursing, University of Illinois College of Nursing, Chicago, IL, USA

## Abstract

**Background:**

Patients with obesity, diabetes, and chronic kidney disease (CKD) are generally physically inactive, have a high mortality rate, and may benefit from an exercise program.

**Methods:**

We performed a 24-week randomized controlled feasibility study comparing aerobic exercise plus optimal medical management to medical management alone in patients with type 2 diabetes, obesity (body mass index [BMI] > 30 kg/m^2^), and stage 2-4 CKD (estimated glomerular filtration rate [eGFR] 15-90 mL/min/1.73 m^2 ^with persistent proteinuria). Subjects randomized to exercise underwent thrice weekly aerobic training for 6 followed by 18 weeks of supervised home exercise. The primary outcome variable was change in proteinuria.

**Results:**

Seven subjects randomized to exercise and 4 control subjects completed the study. Exercise training resulted in an increase in exercise duration during treadmill testing, which was accompanied by slight but insignificant decreases in resting systolic blood pressure and 24-hour proteinuria. Exercise did not alter GFR, hemoglobin, glycated hemoglobin, serum lipids, or C-reactive protein (CRP). Caloric intake and body weight and composition also did not change with exercise training.

**Conclusion:**

Exercise training in obese diabetic patients with CKD is feasible and may have clinical benefits. A large-scale randomized controlled trial to determine the effects of exercise on renal functions, cardiovascular fitness, inflammation, and oxidative stress in diabetic patients with CKD is planned.

## Background

Diabetes mellitus now affects approximately 250 million people worldwide, a figure expected to reach 400 million (approximately 7% of the adult population) by 2025 [1]. This global epidemic of diabetes is in large part due to obesity and sedentary lifestyle. Increasing numbers of patients with diabetic complications will impose an enormous burden on the healthcare system [2,3]. Kidney disease is the most feared complication of diabetes, due to its substantial co-morbidity (need for dialysis, blindness, amputations, etc.), cost, and mortality (the annual mortality rate of diabetic patients with kidney failure on dialysis is about 25%) [4]. The major determinants of kidney disease and its progression to end-stage kidney failure in type 2 diabetes are uncontrolled blood glucose, blood pressure and albuminuria [5-7]. The Steno-2 study provides good evidence that further improvements in outcome can only be achieved through multifactorial intervention, consisting of lifestyle modifications in addition to pharmacologic measures [8].

Regular exercise with [9,10] or without [9,11,12] dietary intervention and/or oral blood glucose-lowering medication [13,14] has been shown to have benefits in patients with Type 2 diabetes. However, most of these studies were done in subjects with newly-diagnosed diabetes without complications and/or only insulin resistance. Current guidelines from the American Diabetes Association (ADA) and the European Association for the Study of Diabetes recognize the value of exercise [15-17]. However, there is no evidence that exercise will prevent or ameliorate complications such as chronic kidney disease (CKD) in this population.

The present study seeks to investigate the hypothesis that increasing energy expenditure by aerobic exercise will have cardiovascular benefits, including decreased blood pressure, decreased heart rate, and increased exercise tolerance, and will result in a decrease in proteinuria in obese diabetic patients with CKD. We also wished to determine if exercise would result in weight loss and improved body composition, improved glucose and lipid control, and a decrease in inflammation.

## Methods

### Study Design

This was a 24-week randomized controlled pilot study. After baseline screening, subjects were randomized to an exercise or control group. Subjects randomized to exercise underwent thrice weekly training for 6 weeks in the research exercise laboratory at Hines VA Hospital, followed by 18 weeks of supervised home exercise. Both groups received standard of care medical treatment for diabetes and CKD, including diabetes education. The primary outcome variable was change in proteinuria. Exercise training parameters (resting and maximal blood pressure, heart rate, and oxygen consumption), hemoglobin, glycated hemoglobin, lipid profile, C-reactive protein (CRP), dietary caloric intake, and body weight and composition were secondary outcome variables. This research was carried out in compliance with the Helsinki Declaration and was approved by the Hines VA IRB. Written informed consent was obtained from each participant.

### Study Sample

Our study sample consisted of 20 subjects from the renal outpatient clinic of Hines VA Hospital with type 2 diabetes, obesity (BMI > 30 kg/m^2^), and stage 2-4 CKD (eGFR 15-90 mL/min/1.73 m^2 ^with persistent proteinuria, i.e. urine protein/creatinine > 200 mg/g for ≥ 3 months) (see Table 1 for inclusion and exclusion criteria).

**Table 1:** Study Inclusion and Exclusion Criteria

Inclusion Criteria	Exclusion Criteria
CKD Stages 2-4	CKD stages other than 2-4
BMI ≥ 30	Hyperparathyroidism/osteoporosis
Diabetes mellitus	Symptomatic neuropathy/retinopathy
Proteinuria (urine protein/creatinine > 200 mg/g for ≥ 3 months)	Positive stress test due to coronary arterial disease
Treatment with ACE inhibitor or ARB, aspirin, and statin (if LDL > 100)	Symptomatic cardiovascular disease
	Congestive heart failure (New York Heart Association Class III or IV)
	Chronic obstructive pulmonary disease (FEV1 < 50% predicted and/or requires supplemental oxygen support during exercise)
	Complaints of angina during the stress test
	Cerebrovascular disease/cognitive impairment
	Renal transplant
	Inability to walk on the treadmill
	Any unforeseen illness or disability that would preclude exercise testing or training
	Participation in a formal exercise program within the previous 12 weeks

BMI = body mass index (kg/m^2^), FEV1 = forced expiratory volume in one second; ACE = angiotensin converting enzyme; ARB = angiotensin receptor blocker; LDL = low density lipoprotein

### Study Procedures

All subjects underwent a full history and physical examination and an electrocardiogram (ECG). They then underwent a symptom-limited, treadmill, exercise stress test. If the stress test was negative, the patient was continued in the study and asked to complete questionnaires, nutritional assessment, and laboratory tests. After all baseline data were obtained, if patients were still eligible, they were randomized using a 2 × 2 block randomization scheme to the exercise or control group. The above testing was repeated after 6 weeks (at the end of the training period) and 24 weeks (end of study).

### Aerobic Training Protocol

Individuals assigned to the exercise group received instruction in walking and proper walking shoe selection. An introductory session was completed to educate the patient on developing a walking program, familiarize him/her with the lab and practice walking on treadmill. Each exercise training session included 3 to 5 minutes of warm-up, range-of-motion exercises, interval training, cool-down, and post-exercise range-of-motion exercises. Each subject's training program was individualized and based on the results of the baseline exercise tests. Exercise intensities listed in Table 2 served as a guide for exercise prescription. Total exercise time began at 30 minutes and gradually increased by 5 minutes every two weeks. Patients were not allowed to exercise at a heart rate beyond that achieved on the maximal exercise test. Training was completed on the treadmill, outside (weather permitting) or in the research building corridors. After six weeks of supervised training, subjects were expected to continue their walking program unsupervised in their community and to increase their step count/structured walk by 10% each week. Study staff called them weekly to ask about step counts and their walking program.

**Table 2:** Exercise Training Schedule

Weeks	Light (25-44% VO_2 _peak)	Moderate (45-59% VO_2 _peak)	Hard (60-84% VO_2 _peak)	Very Hard (>85% VO_2 _peak)
1-3	20% (6 min)	60% (18 min)	20% (6 min)	0%
4-6	15% (6 min)	55% (22 min)	30% (12 min)	0%

Percent time spent at a given exercise training intensity (% peak VO_2_) for exercise group (approximate minutes at each intensity are in parenthesis). Values expressed in the table are a percentage of the total workout time (e.g., 10 min of a 30 minute workout = 33%). Total exercise time begins at 30 minutes and increases by 5 minutes every two weeks until 40 minutes is achieved while supervised.

### Exercise-induced Hypoglycemia

To prevent exercise-induced hypoglycemia, patients were exercised one to two hours after a meal (generally after breakfast). A fingerstick blood glucose was performed prior to exercising; if the glucose was <110 mg/dL, 30 g of carbohydrate was administered prior to exercising.

### Control group protocol

Control patients underwent the same testing battery but did not participate in any exercise training.

### Specific Methods

#### Symptom-limited treadmill test protocol

Subjects exercised on a treadmill (Marquette Series 2000 treadmill) attached to an ECG monitor. Exercise began at 0% grade and a speed of 1.8 mph. Increases in percent grade occurred every 30 seconds, and, after the first six minutes, speed increased every three minutes. The following measurements were made during all exercise tests. Oxygen uptake was determined using the MedGraphics CPX/MAX/D™ system for breath by breath analysis (St. Paul, MN). Prior to each test, the analyzers were calibrated with reference gases and room air. Heart rate was derived from a standard ECG. A Marquette CASE (Cardiac Assessment System for Exercise Testing) system was used for continuous visual monitoring and recording ECG. A 12-lead ECG was taken every minute during all exercise tests. Blood pressure (BP) was determined by the auscultatory technique, using a sphygmomanometer, a pre-gauged adult cuff, and a stethoscope. The first and last Korotkoff sounds were recorded. BP was taken pre-exercise, every two minutes while the subject was exercising, and every minute post-exercise until the patient's BP approached baseline values.

#### Nutritional and Body Composition Assessment

Subjects reported a food record at baseline and then were asked to keep 6 non-consecutive days of food records during the first 6 weeks and then every 3 weeks during weeks 7-24. Average daily intake in kilocalories (kcal) was calculated using Food Processor SQL Edition (Version 9.5.0, ESHA Research, Salem, Oregon, Copyright 2004). In addition, the Harris-Benedict equation (HBE) with appropriate adjustment for activity level was used to estimate energy requirements (kcal) for each subject.

Assessment of body fat/composition was measured by air displacement plethysmography (ADP) (BOD POD, Life Measurement, Inc., Concord, CA USA) [18]. After body volume was determined, thoracic gas volume was determined in order to allow determination of percentage lean and fat weight. The BOD POD is analogous to underwater weighing without having to go under water (air displacement is used instead of water displacement) and has been used in subjects up to 500 pounds with high accuracy.

**Laboratory values **measured included serum urea nitrogen and creatinine, urea and creatinine clearances (to calculate glomerular filtration rate), random urine albumin/creatinine and protein/creatinine ratios, 24-hour urine protein excretion, hemoglobin, glycated hemoglobin, lipid profile, and C-reactive protein (CRP). These tests were done at baseline, at the end of exercise training (6 weeks), and at study completion (24 weeks).

### Data Analysis

We performed within-group and between-group comparisons using mixed-effect linear regression models that included indicators for group (control vs. intervention), time (in weeks), and an interaction term (group × time). To address the possible correlation of an individual's measures over time, the models included a random effect. For analytic purposes, urine protein data were log transformed in order to normalize the data. A P-value of < 0.05 was taken to be significant.

## Results

Twenty patients were recruited and signed the consent form. One subject did not qualify based on screening laboratory tests. Of the remaining 19 subjects, 6 were dropped from the study due to a positive stress test (2 patients), investigator decision (2 patients), patient wish (1 patient), and commencement of hemodialysis (1 patient). Thirteen subjects were then randomized, 7 to the exercise group and 6 to the control group. Two of the 6 control subjects were dropped subsequent to randomization and prior to any testing (due to investigator decision and patient wish, respectively), leaving 4 subjects in the control group. All subjects were male; mean age was 66 (range 55-81).

**Figure 1:**
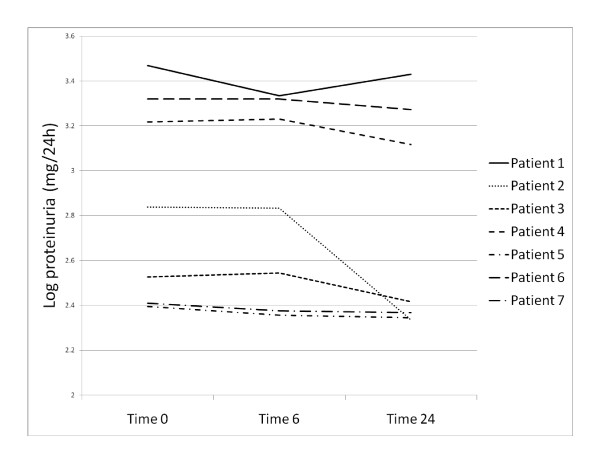
Plot depicting effect of exercise on proteinuria in 7 subjects undergoing exercise training (structured exercise training for 6 weeks followed by home exercise for 18 weeks)

Data for the exercise and control group patients are given in Tables 3 and 4. Thrice weekly exercise training for 6 weeks followed by 18 weeks of home exercise resulted in an increase in exercise duration during treadmill testing at 6 weeks, which persisted until 24 weeks. There was a tendency for oxygen consumption at isotime during the treadmill testing to decrease (isotime is defined as a time when workload was the same in all subjects), which was accompanied by a slight decrease in resting systolic blood pressure, again suggesting a training effect. There was also a trend toward a decrease in 24-hour proteinuria (Figure 1). However, there were no significant between-groups effects over time for exercise duration, oxygen consumption at isotime, blood pressure, or 24-hour proteinuria, probably due to the small sample size. Six weeks of exercise training did not affect GFR nor improve hemoglobin or biochemical parameters such as glycated hemoglobin and serum lipids, and did not diminish CRP (as a marker of inflammation). Caloric intake and body weight and composition also did not change with exercise training.

**Table 3:** Data in Subjects Randomized to Exercise

Exercise Parameters	Baseline	6 weeks	24 weeks
Exercise duration (min)	6.6 ± 2.2	11.3 ± 2.4	10.2 ± 2.8
Maximal oxygen consumption (mL/kg/min)	14.9 ± 1.1	13.8 ± 3.9	15.6 ± 2.4
Oxygen consumption (isotime) (mL/kg/min)	11.9 ± 1.7	10.0 ± 0.7	9.8 ± 1.4
Resting heart rate (bpm)	80 ± 16	82 ± 22	81 ± 21
Maximum heart rate (bpm)	116 ± 16	121 ± 14	118 ± 17
Resting systolic blood pressure (mmHg)	130 ± 28	132 ± 13	113 ± 16
Resting diastolic blood pressure (mmHg)	71 ± 20	71 ± 11	65 ± 10
Maximum systolic blood pressure (mmHg)	182 ± 16	188 ± 24	165 ± 21
Maximum RPP#	21094 ± 3475	22616 ± 1983	19206 ± 1402
Mean ± SD # rate pressure product = heart rate × systolic blood pressure			
			
Renal Function Parameters	Baseline	6 weeks	24 weeks
			
Serum creatinine (mg/dL)	2.9 ± 1.5	2.7 ± 1.3	2.8 ± 1.2
Serum urea nitrogen (mg/dL)	64 ± 40	58 ± 48	57 ± 34
Creatinine clearance (mL/min)	64 ± 54	62 ± 40	51 ± 26
Urea clearance (mL/min)	24 ± 20	28 ± 22	26 ± 19
Glomerular filtration rate (mL/min)#	44 ± 36	45 ± 31	39 ± 22
Urine albumin/creatinine (mg/g)	327 ± 385	242 ± 260	305 ± 456
Urine protein/creatinine (mg/g)	565 ± 600	419 ± 417	493 ± 544
Urine protein excretion (mg/24 h)	1020 ± 1081	891 ± 831	821 ± 1010
# (Creatinine clearance + urea clearance)/2			
			
Biochemical Parameters	Baseline	6 weeks	24 weeks
			
Hemoglobin (g/dL)	12.7 ± 2.1	12.7 ± 2.2	12.5 ± 1.8
Total cholesterol (mg/dL)	125 ± 25	116 ± 17	128 ± 25
Low density cholesterol (mg/dL)	59 ± 17	51 ± 16	54 ± 11
High density cholesterol (mL/min)	38 ± 6	37 ± 5	39 ± 7
Triglycerides (mg/dL)	137 ± 45	144 ± 60	179 ± 89
Glycated hemoglobin (%)	7.4 ± 1.1	7.5 ± 1.2	8.3 ± 2.4
C-reactive protein (mg/dL)	0.55 ± 0.72	0.72 ± 0.99	0.89 ± 0.92
			
Nutrition/Body Composition Parameters	Baseline	6 weeks	24 weeks
			
Calorie intake (kcal/day)	1878 ± 563	2011 ± 396	1939 ± 656
Body weight (kg)	116 ± 27	116 ± 26	115 ± 23
Fat weight (%)	40 ± 3	40 ± 3	40 ± 4
Lean weight (%)	60 ± 3	60 ± 3	60 ± 4

**Table 4:** Data in Control Subjects

Exercise Parameters	Baseline	6 weeks	24 weeks
Exercise duration (min)	4.3 ± 1.1	5.5 ± 3.2	6.6 ± 2.1
Maximal oxygen consumption (mL/kg/min)	10.9 ± 1.0	11.8 ± 2.0	11.9 ± 1.3
Oxygen consumption (isotime) (mL/kg/min)	9.1 ± 0.3	9.0 ± 0.2	9.8 ± 0.5
Resting heart rate (bpm)	75 ± 16	76 ± 10	70 ± 19
Maximum heart rate (bpm)	112 ± 16	107 ± 16	105 ± 13
Resting systolic blood pressure (mmHg)	157 ± 14	156 ± 23	136 ± 5
Resting diastolic blood pressure (mmHg)	83 ± 20	77 ± 12	77 ± 8
Maximum systolic blood pressure (mmHg)	207 ± 28	188 ± 47	184 ± 22
Maximum RPP#	23219 ± 6046	20939 ± 7809	19373 ± 4215
Mean ± SD # rate pressure product = heart rate × systolic blood pressure			
			
Renal Function Parameters	Baseline	6 weeks	24 weeks
			
Serum creatinine (mg/dL)	2.3 ± 0.1	2.1 ± 0.2	2.1 ± 0.4
Serum urea nitrogen (mg/dL)	45 ± 11	48 ± 12	50 ± 18
Creatinine clearance (mL/min)	66 ± 19	54 ± 22	64 ± 10
Urea clearance (mL/min)	28 ± 0.2	18 ± 5.7	19 ± 0.5
Glomerular filtration rate (mL/min)#	47 ± 9.5	36 ± 14	41 ± 5.3
Urine albumin/creatinine (mg/g)	156 ± 148	162 ± 173	221 ± 304
Urine protein/creatinine (mg/g)	347 ± 178	305 ± 186	387 ± 374
Urine protein excretion (mg/24 h)	542 ± 258	383 ± 256	490 ± 237
# (Creatinine clearance + urea clearance)/2			
			
Biochemical Parameters	Baseline	6 weeks	24 weeks
			
Hemoglobin (g/dL)	11.8 ± 1.9	12.0 ± 1.8	11.9 ± 1.3
Total cholesterol (mg/dL)	152 ± 38	160 ± 53	146 ± 31
Low density cholesterol (mg/dL)	75 ± 24	80 ± 41	69 ± 22
High density cholesterol (mL/min)	33 ± 6	31 ± 3	31 ± 4
Triglycerides (mg/dL)	223 ± 92	245 ± 44	225 ± 104
Glycated hemoglobin (%)	8.2 ± 1.7	8.7 ± 2.6	8.1 ± 3.7
C-reactive protein (mg/dL)	1.7 ± 2.4	0.73 ± 0.32	1.17 ± 0.71
			
Body Composition Parameters	Baseline	6 weeks	24 weeks
			
Calorie intake (kcal/day)	1869 ± 13	2165 ± 658	2192 ± 537
Body weight (kg)	140 ± 15	139 ± 15	136 ± 20
Fat weight (%)	50 ± 5	53 ± 3	50 ± 5
Lean weight (%)	50 ± 5	47 ± 3	50 ± 5

## Discussion

To our knowledge, this is the first randomized controlled trial that has examined the effects of exercise training in diabetic patients with CKD. However, there have been a few previous studies that have examined the effects of exercise on renal function in primarily non-diabetic CKD patients. Boyce et al. found, in a crossover-design trial, that four months of exercise training, via stationary cycling, in sixteen non-dialysis CKD subjects (of whom 8 completed the trial) decreased blood pressure (systolic and diastolic), increased peak oxygen consumption, but had no effect on declining GFR [19]. In another non-randomized trial, Clyne et al. found that exercise training for 3 months via bicycle ergometry in 10 predialytic CKD patients increased maximal exercise capacity and decreased heart rate at equal load and increased thigh muscular function assessed by static and dynamic endurance, but these benefits were not associated with improved hemoglobin, GFR, blood pressure, or echocardiographic findings [20]. In a randomized study of 30 patients with moderate CKD, Eidemak et al. found that aerobic exercise increased maximum work capacity but had no effect on declining GFR over a 20-month observation period [21]. The lack of effect of land-based exercise on renal function is consistent with experimental findings in rats with renal mass reduction, in which treadmill exercise for 60 days did not improve GFR [22]. In distinction to these primarily negative studies, Pechter et al. reported that a 12-week low-intensity aquatic exercise program in 26 patients with mild to moderate CKD resulted in decreased blood pressure, decreased proteinuria, and a slight improvement in GFR. This was associated with a decrease in systolic and diastolic blood pressure and oxidative stress indices (lipid peroxidation products and reduced glutathione) [23]. Aquatic exercise may thus decrease proteinuria and improve GFR, as was shown previously in rats with subtotal nephrectomy [24]. However, the results of this study must be interpreted with caution due to its non-randomized design. Moreover, whether or not the putative salutary effects of aquatic exercise can be extended to land-based aerobic exercise is unclear, as water immersion is known to improve renal function, probably due to an improvement in renal hemodyanamics associated with decreases in vasopressor and increases in vasodepressor hormones [25].

Diabetic patients with CKD and overt albuminuria typically develop progressive kidney failure [7]. Therefore, the effects of exercise on albuminuria and progression of kidney disease in this population is of great clinical importance. Albuminuria in type 2 diabetic patients is associated with endothelial dysfunction and low-grade inflammation [26]. Exercise is associated with a decrease in inflammation, and there is a positive association between inflammation and microalbuminuria [27]. Thus an exercise-induced reduction in inflammation and/or oxidative stress might be expected to decrease proteinuria. Cytokines and inflammatory markers may directly mediate glomerular and renal damage leading to albuminuria and renal failure [28]. We did not find significant changes in proteinuria or C-reactive protein in our study, but this can probably be explained by the substantial intra-patient variability and small sample size.

We also did not find any changes in glycated hemoglobin, lipid profile, or body weight or composition. Although all subjects did receive nutritional counseling, they did not specifically undergo caloric restriction or other forms of dietary modification. It is likely that a more prolonged and intensive exercise training program or an exercise program coupled with calorie restriction would have been required to result in weight loss and a decrease in fat mass. In this regard, long-term (1 year) aerobic coupled with resistance training has been reported to improve these parameters in type 2 diabetic patients without kidney disease and only mild obesity (BMI 30) [13]. Renal function and proteinuria were not assessed in that study.

## Conclusion

In summary, we have demonstrated in a pilot study that exercise training in obese diabetic patients with CKD is feasible and may have clinical benefits. A larger-scale randomized controlled trial to determine the effects of exercise on renal functions, cardiovascular fitness, inflammation, and oxidative stress in diabetic patients with CKD is planned.

## Competing interests

The authors declare that they have no competing interests.

## Authors' contributions

DJL was responsible for the overall design and supervision of the study and manuscript preparation. IM helped to design the study and assisted in manuscript preparation. JPB and SQ enrolled patients and supervised exercise tests. CSJ was responsible for exercise training and data management. CC carried out the dietary studies. LCE served as the cardiology consultant. BMS did statistical analyses. EGC was responsible for administration of the research exercise laboratory and assisted in manuscript preparation. All authors read and approved the final manuscript.
